# Semi-Annual Meeting of the Association of Veterinary Science and Comparative Medicine

**Published:** 1887-04

**Authors:** H. F. James

**Affiliations:** Sec’y


Secretary

---

### SEMI-ANNUAL MEETING OF THE ASSOCIATION OF VETERINARY SCIENCE AND COMPARATIVE MEDICINE.

The third semi-annual meeting of the Missouri State Association of Veterinary Science and Comparative Medicine, was held at the State University, Columbia, Mo., Dec. 6th. The following members were present: Paul Paquin, State Veterinarian, Columbia; T. E. White, D. V. S., Sedalia; James Johnston, V. S., St. Joseph; A. Ronif, V. S.; H. B. Platt, V. S., and H. F. James, V. S., St. Louis. The association was complimented by the presence of Profs. McAlister and Moss, members of the medical faculty of the University. Balloting for officers for the ensuing year resulted in the re-election of Paul Paquin as president, T. E. White, 1st vice-president; Jas. Johnston, 2d vice-president; H. B. Adair, treasurer; H. F. James, secretary; H. B. Platt, C. W. Crowley, A. Ronif, censors. Prof. McAlister delivered a short and impressive speech on the importance of comparative medicine. H. F. James, of St. Louis, read a paper on pleuro-pneumonia, in which he quoted some words from a letter which he had lately received from Prof. Smith, of the Ontario Veterinary College, Toronto, Canada, to the effect that the professor believed we would be forced to resort to inoculation to successfully combat pleuro-pneumonia, and stating that from what he had seen of it in Edinburgh with Mr. Rutherford, its results were beyond the range of speculation. This opinion coming from such a widely known and eminently practical authority, carried with it due weight. The discussion which followed shifted to tuberculosis, on which subject Dr. Adair, of Kansas City, was to have read a paper. Dr. T. E. White, of Sedalia, in answer to some inquiries of Drs. McAlister and Moss, gave an instance of the transmission of consumption or tuberculosis from a diseased cow to two children who died within one year of each other. Post-mortems on the children showed tubercular lesions. There was no consumptive taint on either the maternal or paternal side as far back as the family records went. The father, who was a physician, disbelieved both in the existence of tuberculosis in cows and its transmissibility to the human being through the milk, and, although warned by a veterinarian that his family milch cow was tuberculous, would not part with her until the loss of his children forced him to order her destruction. A loathsome mass of tubercle was found in the chest, and the childless man was overwhelmed with grief at the sad results of his obstinacy. There are any number of cases equally conclusive.

Dr. Paul Paquin gave some very interesting points gleaned in his recent trip to France. The laboratory which he is getting into working order at Columbia, cannot but be of the greatest practical benefit to the State, and its utility is endorsed by every member of the association.

H. F. JAMES, Sec'y.

